# The Venom of Scorpions

**Published:** 1911-01-28

**Authors:** 


### Tropical Medicine.

### THE VENOM OF SCORPIONS.

There are many different varieties of scorpions in different tropical countries. The great majority of them are poisonous, some, indeed, fatally so, death being due to the action of their toxins upon the respiratory centre. Probably the account given by Dr. W. H. Wilson, of the Egyptian Government School of Medicine, in his report on the venom of scorpions, applies with slight modification to other countries also. The three common species in Egypt are *Buthus quinquestriatus*, *Prionurus citrinus*, and *Buthus maurus*. The chief symptoms which result from a bite from these are local redness and swelling, sometimes no more than that which would be caused by a mosquito bite, accompanied in severe cases by hallucinations and mental disturbance, sometimes convulsions, weak pulse, rapid respiration, and possibly death from asphyxia.

### Scorpion Poison.

The venom is a clear, slightly viscid fluid of faintly acid reaction and high specific gravity. It contains from 20 to 28 per cent. of solids, of which inorganic salts form a considerable part. Proteids, in part coagulable by heat, are present, and probably form the most important organic constituent. The characters of the venom appear to differ in different species. The active principle is apparently of a proteid nature. So far investigations tend to show that it is either a nucleo-albumin, an acid-albumin, or a primary albumose. It is soluble in glycerin and in saline solution, probably insoluble in pure water, and insoluble in 85 per cent. alcohol. It is unaffected by drying. When in solution it is very resistant to putrefaction, and it is destroyed by heating to 100° C. for 12 to 15 minutes. The minimum fatal dose of the toxin for the guinea-pig is approximately 1 mgm. per kilogramme. The toxic value is therefore 10,000,000. The amount of toxin contained in the glands of different species of scorpion appears to differ. Of three species examined, most could be obtained from *Buthus quinquestriatus*, least from *Prionurus citrinus*; the former is therefore to be regarded as the most venomous.

### Symptoms Produced by a Sting.

The symptoms produced by the venom in animals are referable to the muscular and glandular tissues, consisting of muscular spasms, copious secretions, and apparent muscular paralysis. The muscular spasms are of peripheral origin and are due to the direct exciting action of the poison and to the increased effect of stimuli upon the contractile tissue. The paralysis is due to the great depression in the excitability of the muscles to direct, and especially indirect excitation resulting from the action of the poison. Death is mainly due to the inability of the respiratory muscles to respond to stimuli reaching them from the central nervous system. There also appears to be a direct action on the pulmonary tissues, the effect of the poison being possibly to produce spasm of the smooth muscle of the bronchi and pulmonary blood-vessels. The effects of the scorpion venom in man are comparable to those seen in the guinea-pig, the convulsive symptoms being, however, much less prominent. Local pain, vomiting, sweating, and salivation are the most marked symptoms.

### Treatment.

Death from scorpion sting occurs chiefly amongst children; it occasionally occurs in adults, but it is unusual above the age of 15. Certain animals living under conditions which render them liable to the attack of scorpions are immune to the venom.

Nicolle and Catouillard experimented with the venom of *Heterometrus maurus*, a common North African species, with a view to finding an antidote or a protective serum, but their results were disappointing. Antivenin, as is to be expected, has no protective action, as scorpion poison does not seem to resemble any form of snake poison. Some persons, fakirs and others, have a curious power of handling scorpions with impunity, although stung by them, so that there must be some way of producing immunity. The treatment most recommended is the immediate application of a paste of ipecacuanha.

